# Numerical Simulation of Output Response of PVDF Sensor Attached on a Cantilever Beam Subjected to Impact Loading

**DOI:** 10.3390/s16050601

**Published:** 2016-04-27

**Authors:** Cao Vu Dung, Eiichi Sasaki

**Affiliations:** Department of Civil Engineering, Tokyo Institute of Technology, 2-12-1 Ookayama, Meguro-ku, Tokyo 152-8552, Japan; sasaki.e.ab@m.titech.ac.jp

**Keywords:** numerical simulation, PVDF sensor, impact test, cantilever beam

## Abstract

Polyvinylidene Flouride (PVDF) is a film-type polymer that has been used as sensors and actuators in various applications due to its mechanical toughness, flexibility, and low density. A PVDF sensor typically covers an area of the host structure over which mechanical stress/strain is averaged and converted to electrical energy. This study investigates the fundamental “stress-averaging” mechanism for dynamic strain sensing in the in-plane mode. A numerical simulation was conducted to simulate the “stress-averaging” mechanism of a PVDF sensor attached on a cantilever beam subjected to an impact loading, taking into account the contribution of piezoelectricity, the cantilever beam’s modal properties, and electronic signal conditioning. Impact tests and FEM analysis were also carried out to verify the numerical simulation results. The results of impact tests indicate the excellent capability of the attached PVDF sensor in capturing the fundamental natural frequencies of the cantilever beam. There is a good agreement between the PVDF sensor’s output voltage predicted by the numerical simulation and that obtained in the impact tests. Parametric studies were conducted to investigate the effects of sensor size and sensor position and it is shown that a larger sensor tends to generate higher output voltage than a smaller one at the same location. However, the effect of sensor location seems to be more significant for larger sensors due to the cancelling problem. Overall, PVDF sensors exhibit excellent sensing capability for in-plane dynamic strain induced by impact loading.

## 1. Introduction

Polyvinylidene Fluoride (PVDF) is a thin film-type polymer that is mechanically tough, flexible, and low density. The piezoelectric effect in elongated and polarized films of polymers, particularly of PVDF, was discovered by Kawai [1] in 1969. Since then, the fundamental properties of PVDF have been extensively investigated. The semi-crystalline molecular structure of PVDF consists of long chain molecules with a repeating CF2CH2 unit. Application of heat, electrical fields, and pressure can interconvert the four different forms of PVDF’s crystalline domains [2,3,4]. The molecular dipoles in the crystalline parts are oriented by thermal poling or corona poling, thus resulting in a permanent polarization. In the β-phase, PVDF exhibits piezoelectric effect which means mechanical energy can be converted to electrical energy and vice versa. Therefore, PVDF has been frequently used to manufacture sensors and actuators for a number of practical applications such as shock impact and pressure sensors [5,6,7,8], biomedical [9,10,11], acoustic [12,13,14,15], tactile sensors [16,17,18], active vibration control [19], and structural health monitoring of civil and aerospace structures [20,21,22].

Many previous studies have focused on the use of PVDF sensors in the out-of-plane (3-3) mode in which mechanical stress is induced in the thickness (poled) direction. However, the in-plane sensing mode of PVDF sensor has also been frequently employed for dynamic impact sensing. Lee and O’Sullivan [23] developed a uniaxial strain rate gage that measures only strain rate along a specified direction by combining the effective surface electrodes, appropriate skew angle, and the correct polarization profile. Wang and Wang [24] presented a theoretical approach for feasibility analysis of the application of PVDF sensors to cantilever beam modal testing. Sirohi and Chopra [25] investigated the behavior of piezoelectric elements including piezoceramic (PZT) and piezofilm (PVDF) as dynamic strain sensors of which superior performance compared to conventional strain gages in terms of sensitivity and signal-to-noise ratio was demonstrated. Correction factors to account for transverse strain and shear-lag effect due to bonding layer were analytically derived and experimentally validated by the same authors. Ma *et al.* [26] investigated the effects of a PVDF sensor’s area and the use of a charge amplifier on the measurement capability of a PVDF sensor attached to a cantilever beam that is subjected to impact loading. PVDF sensors proved to be capable of capturing most of the resonant frequencies from transient responses, and their sensitivity was demonstrated to be better than that of conventional strain gages. Kotian *et al*. [27] presented an analytical investigation on the effects of stress-averaging for both in-plane sinusoidal stress waves and in-plane impact-induced stresses. It was concluded that the error induced by stress averaging becomes more significant as sensor length increases, density of structure’s material increases, and magnitude of input stress increases, although the error induced by stress averaging is minimal for most practical applications. Furthermore, only very high frequencies (in the order of kHz) can cause a significant reduction in a PVDF sensor’s output voltage due to stress-averaging.

In civil engineering structures, dynamic strain is one of the most fundamental measures. To capture dynamic strain, conventional foil strain gages have been frequently used. PVDF sensor with its superior signal-to-noise ratio can be an alternative for strain gages in several practical applications. Since strain gage size is usually minute compared to that of host structures, dynamic strain output can be considered strain at a point on the host structural member. On the other hand, PVDF sensors are much larger in size compared to strain gages. An electrode of a PVDF sensor typically covers a relatively large area of the host structural member over which dynamic strain may vary significantly depending on the relative size of the PVDF sensor compared to that of the host member and strain gradient. To understand the mechanism of strain averaging, both extreme scenarios including minor and large strain variations should be investigated. In case of minor strain variation, a strain averaging mechanism was thoroughly discussed in Kotian *et al.* [27] using tensile specimens subjected to sinusoidal excitation. Ma *et al.* [26] indicated that the use of a charge amplifier is indispensable for improvement of a PVDF sensor’s capability in capturing low-frequency vibration modes of a steel cantilever beam subjected to impact loading. However, the conversion mechanism of dynamic strain to output voltage, *i.e.*, strain averaging mechanism, was not explicitly explained in the same study. Moreover, the effect of sensor size was discussed by comparing sensor pairs at a fixed point for the purpose of illustrating charge amplifier’s indispensability for low-frequency measurement. 

This study investigates the strain averaging mechanism of a PVDF sensor attached to an area of the host structural member where large strain variation occurs. A numerical simulation based on the governing equations of piezoelectricity, classical beam theory, and electronic signal conditioning to predict the output voltage of a PVDF sensor would be proposed. To verify the numerical simulation results, experimental impact tests and FEM analysis would be conducted using a steel cantilever beam subjected to impact forces since a cantilever beam can be regarded as the most fundamental and flexible structure that has high strain gradient along its length. The proposed numerical simulation would provide an insight into the mechanism of dynamic impact strain sensing by a PVDF sensor attached to a surface with large strain variation. Furthermore, the effects of sensor size and position on the output voltage would be investigated in parametric studies using the proposed numerical simulation.

## 2. Theoretical Background

### 2.1. Governing Equation for Piezoelectricity

**Figure 1:**
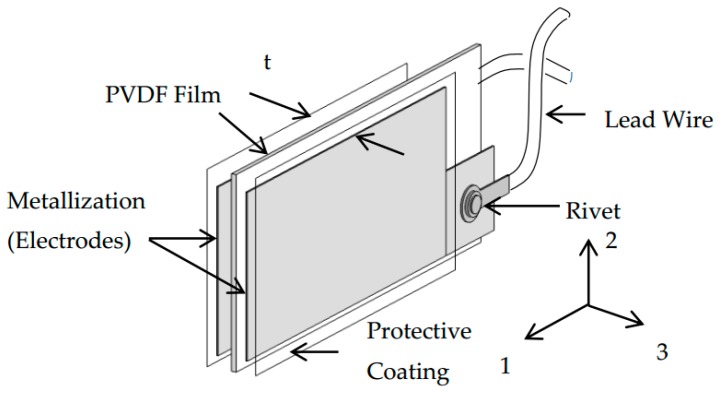
Schematic configuration of PVDF sensor.

Consider the constitutive governing equation that is reduced from the tensor expression for the piezoelectric effect induced by one-dimensional mechanical deformation [28]:

(1) $$D_{3}{=\text{d}}_{31}T_{1}{+\varepsilon }_{33}^{T}E_{3}\;$$

where D_3_ is the electrical displacement component; d_31_ is the piezoelectric constant; T_1_ is the axial stress component, $\varepsilon_{33}^{T}$ is the permittivity component at constant stress, and E_3_ is the electrical field component. Subscripts 1 and 3 indicate the longitudinal direction and the poling direction, respectively (Figure 1). In case the external electrical field is absent (E_3_ = 0), the relation is further reduced to:

(2) $$D_{3}={\text{d}}_{31}T_{1}={\text{d}}_{31}E_{p}S_{1}$$

where $S_{1}$ is the bending strain and E_p_ is the Young’s modulus of the PVDF layer.

The bending strain is expressed by the bending moment/curvature differential equation assuming small deflection and rotation [29]:

(3) $$S_{1}\left(\text{x},\text{ t}\right)=-y_{\text{p}0}\frac{\partial^{2}y\left(\text{x},\text{ t}\right)}{\partial x^{2}}$$

where y_p0_ is the distance from the neutral axis of the cross-section to the center of the PVDF layer; y(x, t) is the transverse displacement of the cantilever beam, which can be represented by a convergent series of the eigenfunctions as

(4) $$y\left(\text{x},\text{ t}\right)=\overset{\infty }{\underset{r=1}{\sum }}\phi_{r}\left(\text{x}\right)\eta_{r}(\text{t})$$

where $\phi_{r}(\text{x})$ is the mass-normalized eigenfunction; $\eta_{r}(\text{t})$ is the modal coordinate of the cantilever beam for the r-th vibration mode. If the cantilever beam is assumed to be proportionally damped, the eigenfunctions denoted by $\phi_{r}(\text{x})$ are the mass-normalized eigenfunctions of the undamped free vibration [30,31].

(5) $$\phi_{r}\left(x\right)=\sqrt{\frac{1}{\text{mL}}}\left[\text{cosh}\frac{\lambda_{r}}{L}\text{x}-\text{cos}\frac{\lambda_{r}}{L}\text{x}-\sigma_{r}(\text{sinh}\frac{\lambda_{r}}{L}\text{x}-\text{sin}\frac{\lambda_{r}}{L}\text{x})\right]$$

where m is the mass per unit length of the cantilever beam; λ_r_ is the dimensionless frequency number for each mode obtained from the following characteristic equation:

(6) 1+cosλcoshλ=0

(7) $$\sigma_{r}=\frac{sinh\lambda_{r}-sin\lambda_{r}}{cosh\lambda_{r}+cos\lambda_{r}}$$

The mass-normalized eigenfunctions satisfy the following orthogonality conditions:

(8) $$\int_{\text{x}=0}^{L}\text{m}\phi_{s}\left(x\right)\phi_{r}\left(x\right)\text{dx}=\delta_{\text{rs}};\int_{\text{x}=0}^{L}\text{EI}\phi_{s}\left(x\right)\frac{d^{4}\phi_{r}\left(x\right)}{{\text{dx}}^{4}}\text{dx}=\omega_{r}^{2}\delta_{\text{rs}}$$

where E is the Young’s modulus of the cantilever beam; δ_rs_ is the Kronecker delta, δ_rs_ = 1 for s = r and δ_rs_ = 0 for s ≠ r; and ω_r_ is the undamped natural frequency of the r-th mode:

(9) $$\omega_{r}=\lambda_{r}^{2}\sqrt{\frac{EI}{{mL}^{4}}}$$

### 2.2. Governing Mechanical Equation

The governing equation of motion can be written as [31]:

(10) $$\text{EI}\frac{\partial^{4}\text{y}(\text{x},\text{ t})}{\partial x^{4}}+{\text{c}}_{s}I\frac{\partial^{5}\text{y}(\text{x},\text{ t})}{\partial x^{4}\partial t}+{\text{c}}_{a}\frac{\partial \text{y}(\text{x},\text{ t})}{\partial t}+\text{m}\frac{\partial^{2}\text{y}(\text{x},\text{ t})}{\partial t^{2}}=\text{p}(\text{t})$$

where y(x, t) is the transverse displacement of the cantilever beam; c_s_ is the equivalent coefficient of strain rate damping; and I is the equivalent area moment of inertia of the cross-section. In terms of damping, c_s_I represents the equivalent damping term of the cross section due to structural viscoelasticity while c_a_ is the viscous air damping coefficient. Both of the above-mentioned damping mechanisms satisfy the proportional damping criterion [30,31]; p(t) is the time history of external excitation. For an impact, p(t) can be represented as:

(11) $$p\left(t\right)=\text{F}\delta (\text{x}-{\text{x}}_{F})\delta (\text{t}-\tau )$$

where F is the magnitude of the impact force; x_F_ is the location of impact force; and δ is the direct delta function.

Substitution of y(x, t) in Equation (4) into the governing equation of motion (Equation (10)) and using the orthogonality conditions (Equation (8)), the modal response of the cantilever beam can be obtained from the following electromechanically coupled ordinary differential equation:

(12) $$\frac{d^{2}\eta_{r}(t)}{{\text{dt}}^{2}}+2\zeta_{r}\omega_{r}\frac{d\eta_{r}(t)}{\text{dt}}+\omega_{r}^{2}\eta_{r}\left(t\right)={\text{N}}_{r}\left(t\right)$$

where $\zeta_{r}$ is the mechanical damping ratio including both effects of strain rate damping and viscous air damping [32],

(13) $$\zeta_{r}=\frac{c_{s}\text{I}\omega_{r}}{2\text{EI}}+\frac{c_{a}}{2\text{m}\omega_{r}}$$

The assumption of proportional damping was discussed in [32]. In this assumption, once the proportionality constants c_s_ and c_a_ are identified using the modal properties (*i.e.*, natural frequencies and damping ratios) of two vibration modes, the other mode’s damping ratio is not arbitrary but mathematically derived from Equation (13). Identified damping ratios of the vibration modes of interest might also be used directly without obtaining the c_s_ and c_a_ values since the resulting electromechanical expressions only need the $\zeta_{r}$ values.

The modal mechanical forcing function, N_r_(t) , can be expressed as,

(14) $$N_{r}\left(t\right)=\int_{\text{x}=0}^{L}\phi_{r}\left(x\right)p\left(t\right)\text{dx}$$

The solution of Equation (12) can be expressed using the unit impulse response function in the form of Duhamel integration as

(15) $$\eta_{r}\left(t\right)=\frac{1}{\omega_{\text{rd}}}\int_{\tau =0}^{t}N_{r}\left(\tau \right)e^{-\zeta_{r}\omega_{r}\left(\text{t}-\tau \right)}\text{sin}\omega_{\text{rd}}(\text{t}-\tau )\text{d}\tau$$

where ω_rd_ is the damped natural frequency of the r-th mode,

(16) $$\omega_{\text{rd}}=\omega_{r}\sqrt{1-\zeta_{r}^{2}}$$

### 2.3. Governing Electrical Equation

There are two equally valid equivalent electrical models of the piezofilm element—one is a voltage source in series with a capacitance that is equal to the capacitance of the sensor, the other a charge generator in parallel with a capacitance [33]. The latter is used in this study. 

Capacitance of a PVDF sensor is expressed as:

(17) $$C_{p}=\varepsilon \frac{A}{t}$$

where ε is the permittivity, which can also be expressed in the form of $\varepsilon =\varepsilon_{r}\varepsilon_{0}$ where $\varepsilon_{r}$ is the relative permittivity (about 12 for PVDF) and $\varepsilon_{0}$ is the permittivity of free space (constant, 8.854 × 10^−12^ F/m); A is the active area of the film’s electrodes; and t is the film thickness.

**Figure 2:**
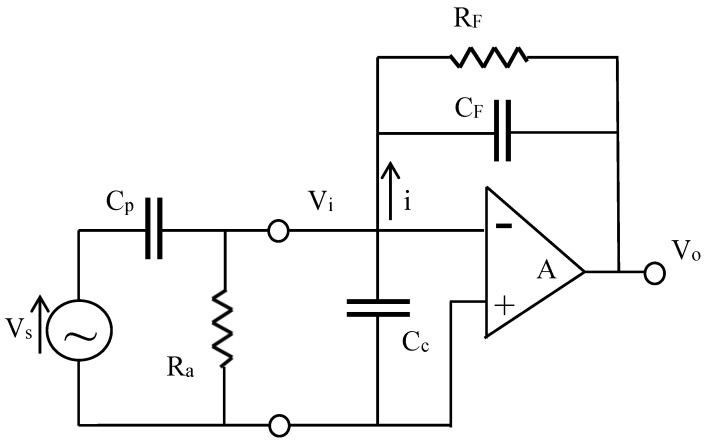
Electrically equivalent model of a PVDF sensor connected to a charge amplifier.

The signal conditioning circuit is shown in Figure 2. The advantages of connecting a PVDF sensor to a charge amplifier have been emphasized in [25]. First, the charge generated by the sensor is transferred onto the feedback capacitance C_F_. The voltage output of a charge amplifier is proportional to the input strain, irrespective of the sensor’s capacitance. The gain is controlled by the feedback capacitance of the charge amplifier. Second, the value of time constant, defined as R_F_C_F_, can be selected in order to navigate the loading effect in order to obtain the desired dynamic frequency range. Ma *et al.* [26] also indicated that a charge amplifier is indispensable for a PVDF sensor, especially a small-sized one, to improve the low-frequency responses of a PVDF sensor.

The governing equation of a charge amplifier’s output voltage can be found in [19]:

(18) $$V_{0}=-\frac{-\text{j}\omega \text{A}{\text{V}}_{s}C_{p}}{\left[\text{j}\omega \left(\left(\text{A}+1\right)C_{F}+{\text{C}}_{p}+{\text{C}}_{c}\right)+(\frac{1}{R_{a}}+(\text{A}+1)\frac{1}{R_{F}})\right]}$$

where ω is the angular frequency (rad/s); V_s_ is the voltage generated by the PVDF sensor; C_p_ is the equivalent capacitance of the PVDF sensor; R_a_ is the output impedance of the PVDF sensor; C_c_ is the equivalent capacitance of the electric wire; A is the gain of the charge amplifier; and C_F_ and R_F_ are the feedback capacitance and impedance of the charge amplifier, respectively.

When the magnitude of gain is large enough, the output voltage equation can be reduced to:

(19) $$V_{0}=\frac{-\text{j}\omega {\text{V}}_{s}C_{p}}{\text{j}\omega {\text{C}}_{F}+\frac{1}{R_{F}}}$$

In the high frequency region, the output voltage can be further reduced to:

(20) $$V_{0}=-\frac{V_{s}C_{p}}{C_{F}}=-\frac{q}{C_{F}}$$

Meanwhile, in the low-frequency region, the amplitude of output voltage is expressed as:

(21) $$\left|V_{0}\right|=-\frac{\omega \text{q}}{\sqrt{\frac{1}{R_{F}^{2}}+\omega^{2}C_{F}^{2}}}$$

where q is the electrical charge accumulated on the PVDF sensor’s electrodes, which is given as

(22) $$\text{q}=\int_{\text{A}}^{}d_{31}E_{p}S_{1}ndA=b_{p}\int_{x_{p1}}^{x_{p2}}d_{31}E_{p}\left(-y_{p0}\frac{\partial^{2}y(x,\;t)}{\partial x^{2}}\right)\text{dx}=-y_{p0}d_{31}E_{p}b_{p}\eta_{r}\left(t\right)\overset{\infty }{\underset{\text{r}=1}{\sum }}\frac{d\phi_{r}\left(x\right)}{\text{dx}}{|}_{x_{p1}}^{x_{p2}}$$

The cut-off frequency is written as:

(23) $$f_{c}=\frac{1}{2\pi {\text{R}}_{F}C_{F}}$$

## 3. Numerical Simulation of PVDF Sensor’s Response to Impact Force

**Figure 3:**
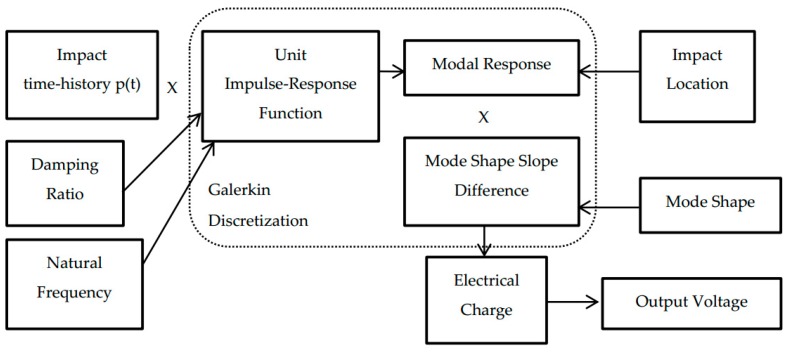
Flow of numerical simulation.

**Figure 4:**
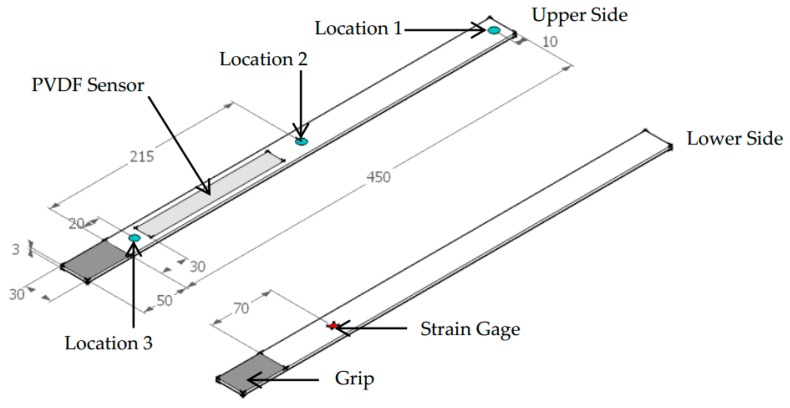
Configuration of cantilever beam with attached sensor and impact locations (Unit: mm).

**Figure 5:**
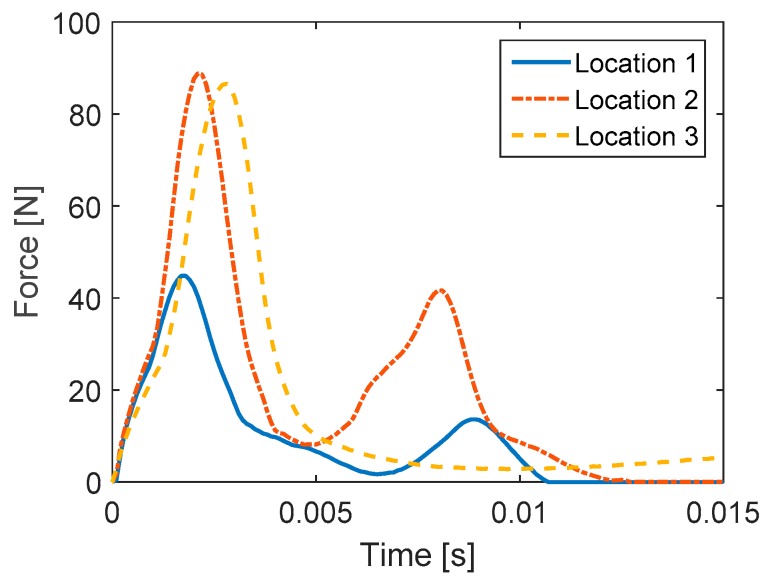
Time-histories of impact loadings at three locations.

Numerical simulation was conducted using MATLAB. The sampling rate was chosen as 10 kHz and one second of the PVDF sensor’s output voltage response was simulated. The basic input parameters for simulation are shown in Table 1. Figure 3 illustrates the simulation flow. Three vibration modes were included for impact at locations 1 and 2 and five modes for location 3. Natural frequencies of the cantilever beam were determined using Equations (9) and (16) for flexural modes. Damping ratios obtained by the half-power method (Table 3) were employed to create the unit impulse-response function. For mode shape simulation, the length increment of 1 mm was chosen. Impact time-histories at three impact locations illustrated in Figure 4 were employed as input impact loading in the simulation process (Figure 5). The unit impulse-response function, modal response, and mode shape slope difference are determined for each vibration mode. The electrical charge is then summed from the contribution of all included vibration modes. Predicted output voltage is finally determined from the electrical charge determined by Equation (20).

## 4. Impact Tests

To obtain the actual impact histories that would be input for numerical simulation and to verify the numerical simulation results, impact tests were conducted by applying impact loadings on a steel cantilever beam on which a PVDF sensor was attached. The piezofilm lab amplifier [34] operating in charge mode was used for signal conditioning. Feedback capacitance of 100 nF was selected. The lower cut-off frequency and higher cut-off frequency were selected as 0.1 Hz and 100 kHz, respectively. The NI USB-4431 enabling four 24-bit simultaneous analog inputs was used for data acquisition. 10 kS/s was chosen for sampling rate and 1 kS/s for data windowing for real-time visualization of the PVDF sensor’s output response during the impact tests. One DT4-028 K/L PVDF sensor [35] was attached to the steel cantilever beam by a commercial double-coated adhesive tape. One edge of the PVDF sensor distances 30 mm from the fixed end of the cantilever beam (Figure 4). The impact hammer PCB 086C03 manufactured by PCB Piezotronics with a mounted plastic tip was used to apply impact force on the cantilever beam at the three impact locations illustrated in Figure 4. During the impact tests, impact signal was transmitted to the ICP sensor signal conditioner provided by PCB Piezotronics. A unit gain was set on the ICP signal conditioner. To verify the numerical simulation results, strain gages were also attached to the opposite side of the cantilever beam to obtain “true” strain (Figure 4). Typical impact force time-histories are shown in Figure 5. Impact force histories at locations 1 and 2 contain two peaks which are both due to the physical contact between the impact hammer and the cantilever beam. Particularly, the first peak represents the first time when the cantilever beam is collided by the hammer tip. The energy absorbed by the cantilever beam is then transformed to displacement of the cantilever beam’s free end, which mainly excites the first vibration mode. Right after the first collision, the hammer tip continues to move in the same direction as that of the cantilever beam’s free end due to the effect of inertia induced by the hammer’s weight and velocity. Approximately one to two milliseconds after the first collision, the hammer hits the tip of cantilever beam the second time, which causes the second impact. However, for impact at location 3 which is close to the fixed end, impact energy absorbed by the cantilever beam is transformed mainly into excitation of higher modes rather than displacement at the point of collision. Therefore, only one peak appears in the time history of impact at location 3. However, time history of impact rather than impact force magnitude (*i.e.*, peak) was employed in the numerical simulation, which means impact force energy is taken into account in the prediction of the PVDF sensor’s output voltage in this study.

## 5. Verification of Numerical Simulation Results by FEM

**Figure 6:**
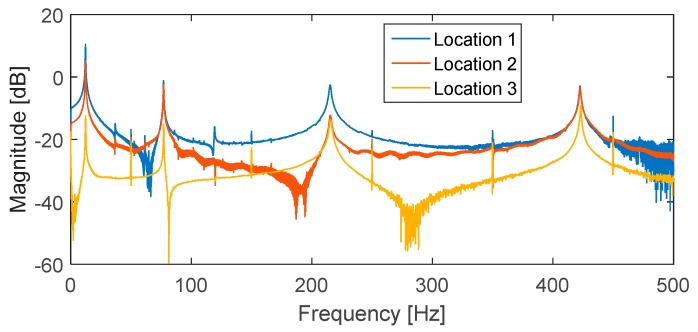
Frequency response functions for three impact locations.

**Figure 7:**
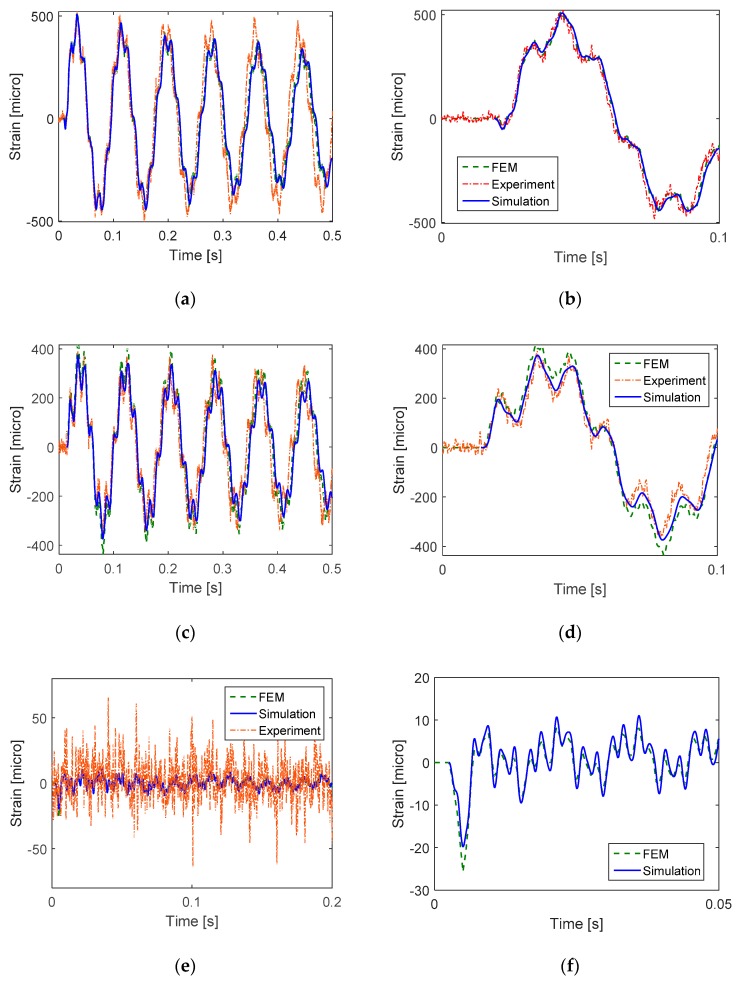
Strain measured at 70 mm from the fixed end for three impact locations. (**a**,**b**) Impact at location 1; (**c**,**d**) Impact at location 2; (**e**,**f**) Impact at location 3.

In order to verify the simulation results, FE model of the cantilever beam subjected to impact loading was analyzed using the commercial software ABAQUS. The purposes of FEM analysis were: (1) To acquire the knowledge of the cantilever beam’s vibration modes, as well as mode shapes and natural frequencies that would be used for the numerical simulation; (2) to obtain dynamic strain for verification of the numerical simulation results; and (3) to convert output impact histories of the ICP signal conditioner into force unit. Table 2 indicates that the natural frequencies of the cantilever beam obtained by FEM analysis, classical beam theory, and modal impact test exhibit a good agreement. Most of the flexural modes can be accurately identified by the PVDF sensor, which indicates the capability of PVDF sensors in identifying natural frequencies of the host structure’s fundamental vibration modes. The first torsional mode at 341.41 Hz did not appear in the frequency response functions (Figure 6), which may indicate the dominance of flexural modes for all three impact locations. If torsional modes are excited due to an impact location that deviates from the central line of the cantilever beam, PVDF sensors can also capture such torsional modes. The superiority of PVDF sensors compared to conventional foil strain gages in capturing torsional modes was indicated in Ma *et al.* [26]. For the second purpose, FEM modal dynamic analysis was conducted using the actual recorded impact that had been previously generated by the impact hammer during the impact tests. Direct damping ratios (Table 3) determined by the half-power point method [36] using the frequencies response functions shown in Figure 6 were used for the first five flexural modes of the cantilever beam in the FEM analysis. To obtain the theoretically simulated strain shown in Figure 7, the transverse displacement in Equation (4) was determined from the mass-normalized eigenfunction written in Equation (5) and the modal coordinate (*i.e.*, the solution of Equation (12)) expressed in Equation (15). Dynamic strain was then calculated in Equation (3) by taking the second derivative of the known transverse displacement. There appears to be a good agreement between the strain values obtained by FEM analysis, those measured in the impact tests and those predicted by the numerical simulation for impacts at locations 1 and 2 (Figure 7a,b). For impact at location 3, only the strain values determined by FEM analysis and those predicted by simulation were compared since the strain amplitude is only as low as the noise level in strain gages. A good agreement was also observed for impact location 3 (Figure 7c).

## 6. Results of Numerical Simulation

**Figure 8:**
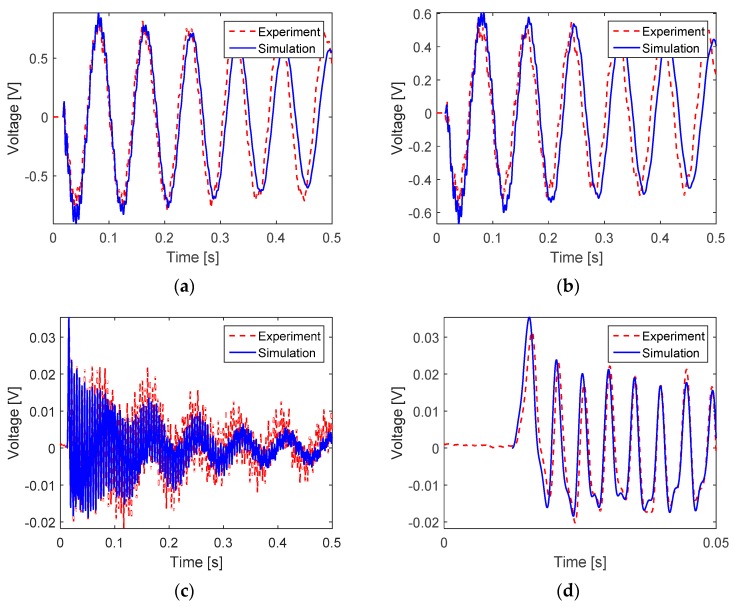
Simulation results for three impact locations. (**a**) Impact at location 1; (**b**) Impact at location 2; (**c**,**d**) Impact at location 3. The sensor size is 155 × 19 × 0.028 (mm). One sensor edge is placed 30 mm from the fixed end of the cantilever beam so that the lead wire goes towards the fixed end.

There appears to be a good agreement between the actual output voltage of the PVDF sensor obtained in the impact test and that predicted by the numerical simulation. Even for impact at location 3 for which the amplitude of output signal is much lower than that for locations 1 and 2 (Figure 8a,b), a reasonable prediction by simulation was also obtained (Figure 8c,d).

**Figure 9:**
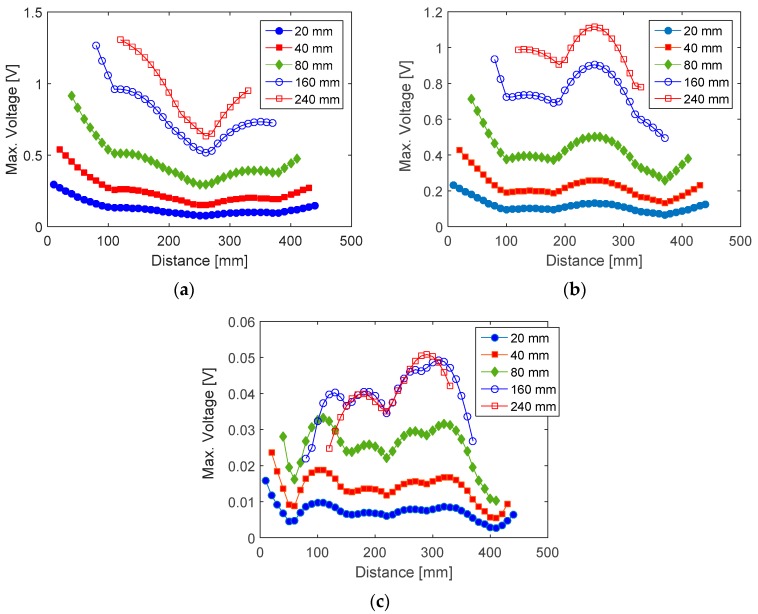
Simulated output voltage of PVDF sensor with different sensor lengths and positions. (**a**) Impact at location 1; (**b**) Impact at location 2; (**c**) Impact at location 3.

**Figure 10:**
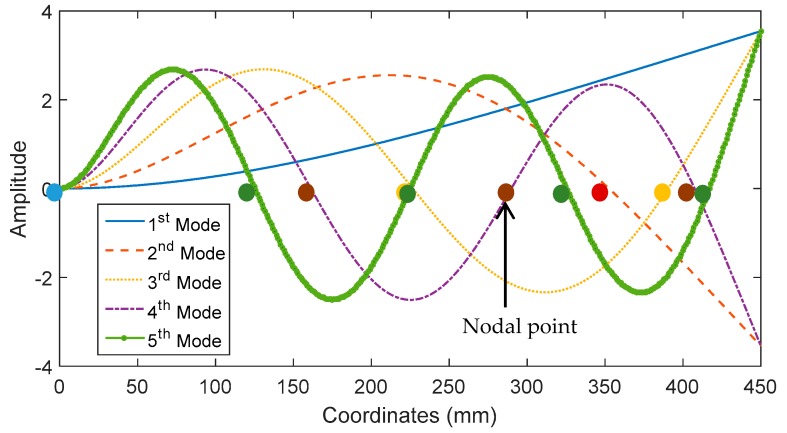
Mode shapes of the first five flexural vibration modes.

Parametric studies were conducted using sensor size and sensor position as input parameters for the numerical simulation described in the previous section in order to investigate the effect of sensor size and sensor position on the output voltage of a PVDF sensor. The input impact forces, damping ratios and all remaining input parameters were chosen similar to those used for the prediction of output voltage shown in Figure 8. Five different sensor lengths including 20, 40, 80, 160, and 240 mm were employed in the simulation. The width of simulated sensors is 20 mm. For the investigation on the effect of sensor position, a midpoint position increment of 10 mm was used. Figure 9 shows the simulated output voltage for different sensor sizes and midpoint positions. For impact at location 1, there appears to be one position at about 260 mm from the fixed end where the output voltage reaches minimum regardless of sensor size. PVDF sensors located closer to the fixed end tend to generate higher peak voltage, which may be attributed to the fact that in the first mode of vibration bending moment increases towards the fixed end. The fact that the minimum voltage occurs at about 260 mm might be due to the problem of cancelling of mode shape slope calculated at the two opposite edges of PVDF sensor (Equation (22)). For impact at locations 2 and 3, the minimum output voltage occurs at multiple positions, which may be due to the cancellation problem at multiple nodal points as a result of higher number of excited vibration modes. The first five flexural mode shapes as well as the corresponding nodal points for each mode shape were illustrated in Figure 10. For all three impact locations, a larger sensor tends to generate higher output voltage and vice versa. For impact location 3, a sensor longer than 240 mm appears to result in no increase in output voltage. Furthermore, larger sensors appear to result in larger variation in the output voltage, which means that the effect of sensor location tends to be more significant for sensor with larger sizes. 

## 7. Conclusions

This study investigates the “stress-averaging” mechanism of the in-plane sensing mode of the PVDF sensor for dynamic strain sensing. A numerical simulation was conducted to predict the output voltage response of a PVDF sensor attached to a steel cantilever beam subjected to impact loading based on the fundamental knowledge of piezoelectricity, classical beam theory, and signal conditioning. FEM analysis and impact tests were also conducted to verify the simulation results. The results of impact test indicate the excellent capability of PVDF sensors in capturing the fundamental natural frequencies of the cantilever beam. The PVDF sensor’s output voltage could be reasonably predicted by the numerical simulation. Parametric studies on the effects of sensor size and sensor position indicate that a larger sensor tends to generate higher output voltage and vice versa. Furthermore, the effect of sensor position seems to be more significant for larger sensors. However, when a large number of modes are excited, e.g., impact at location 3, increasing sensor size may not always result in increased output voltage. The sensor size beyond which increasing sensor size does not result in increased output voltage may depend on the number of dominant vibration modes which in turn depends on impact location. The results indicate that, in order to maximize the signal-to-noise ratio for actual measurements in small scale structures (*i.e.*, the size of an attached PVDF sensor is relatively large with respect to the size of the host structural member), vibration mode shapes of the structural member should be considered to choose the optimal sensor location that helps avoid the cancelling problem. Overall, PVDF sensors exhibit an excellent in-plane sensing capability for dynamic strain induced by impact loading.

## Figures and Tables

**Table 1:** Input parameters for simulation.

Item	Symbol	Parameter	Value
Sensor	L_p_	Length	155 mm
	b_p_	Width	19 mm
	t_p_	Thickness	0.028 mm
	d_31_	Piezoelectric constant	23 × 10^−12^ (C/N)
	E_p_	Young’s modulus	4 GPa
	C_p_	Capacitance	11 nF
Charge Amplifier	f_c_	Cut-off frequency	0.1 Hz
	C_F_	Feedback capacitance	100 nF
Cantilever beam	ρ	Density	7860 kg/m^3^
	E	Young’s modulus	200 GPa

**Table 2:** Natural frequencies of the cantilever beam (Hz).

Mode No.	Mode Type	FEM	Theory	Modal Test			Maximum Difference (%)
				Location 1	Location 2	Location 3	
1	1st Flexural	12.135	12.071	12.29	12.3	12.32	2.02
2	2nd Flexural	76.029	75.651	77.06	77.08	77.15	1.94
3	1st Lateral	120.44	n/a	119.5	n/a	n/a	0.79
4	3rd Flexural	212.89	211.85	215.2	215.4	215.5	1.69
5	1st Torsional	341.41	n/a	n/a	n/a	n/a	n/a
6	4th Flexural	417.28	415.14	422.4	422.5	422.9	1.83
7	5th Flexural	690.06	686.19	698.3	698.3	698.9	1.81
8	2nd Lateral	739.77	n/a	n/a	n/a	n/a	n/a
9	2nd Torsional	1026.7	n/a	n/a	n/a	n/a	n/a
10	6th Flexural	1031.3	1025.1	1044	1044	1044	1.81

**Table 3:** Idenfified damping ratio by half-power method.

	1st Mode	2nd Mode	3rd Mode	4th Mode	5th Mode
Impact at 1	0.011159	0.002566	0.005434	-	-
Impact at 2	0.009391	0.002647	0.006121	-	-
Impact at 3	0.013542	0.002346	0.005920	0.001362	0.002831
